# Factors associated with anxiety and depression in men undergoing fertility investigations: a cross-sectional study

**DOI:** 10.1186/s40359-023-01330-z

**Published:** 2023-09-30

**Authors:** Rim Kooli, Amira Sallem, Dhekra Chebil, Manel Boussabbeh, Bochra Ben Mohamed, Tesnim Ajina, Ines Boughzela, Soumaya Mougou, Meriem Mehdi

**Affiliations:** 1 Laboratory of Cytogenetics and Reproductive Biology, Maternity and Neonatology Center, Fattouma Bourguiba University Teaching Hospital, Monastir, Tunisia; 2 Laboratory of Histology-Embryology and Cytogenetics (LR 18 ES 40), Faculty of Medicine of Monastir, University of Monastir, Monastir, Tunisia; 3 Faculty of Medicine of Sousse, University of Sousse, Sousse, Tunisia; 4 Laboratory for Research On Biologically Compatible Substances, Faculty of Dentistry of Monastir, University of Monastir, Monastir, Tunisia; 5 Psychiatry Service, Fattouma Bourguiba University Teaching Hospital, Monastir, Tunisia; 6 Laboratory of Human Cytogenetics and Reproductive Biology, Farhat Hached Univesity Teaching Hospital, Sousse, Tunisia

**Keywords:** Anxiety, Depression, Male infertility, Semen analysis, Tunisia

## Abstract

**Background:**

Infertility is a real public health issue because of its medical, socio-cultural, and financial impact. It does also have heavy psychological consequences on both partners. This study aimed to assess levels of anxiety and depression among men undergoing infertility investigation and to identify their associated factors.

**Methods:**

We conducted a cross-sectional study in the Laboratory of Cytogenetics and Reproductive Biology of Fattouma Bourguiba University Teaching Hospital (Monastir, Tunisia) between August 30th, 2020, and March 16th, 2021. Anxiety and depression levels were assessed using the valid Arab version of the Hospital Anxiety and Depression scale (HAD). Semen parameters were analyzed and interpreted according to 2021 World Health Organization (WHO) guidelines.

**Results:**

A total of 282 men were included in the current study. The mean HAD-D (depression) and HAD-A (anxiety) scores were of 6.56 ± 3.07 (IQR [4–8]) and 7.94 ± 3.73 (IQR[5–10]) respectively. Univariate analysis showed that patients having two or more comorbidities were nearly five times more likely to be anxious than those without or with only one comorbidity (ORc = 4.71; *p* = 0.007). Furthermore, single patients were about four times more anxious than those in couple having primary or secondary infertility (ORc = 3.85; *p* = 0.027). With regards to semen parameters, patients having hypospermia were more than two times anxious compared with those with normal semen volume (ORc = 2.33; *p* = 0.034). As for depression, we observed that patients with an infertility history lasting for a year or more have a nine times greater risk of depression (ORc = 9.848; *p* = 0.007). With regards to semen parameters, patients exhibiting two or more semen abnormalities, teratozoospermia and increased MAI were more depressed (ORc = 2.478; *p* = 0.036; ORc = 2.549: *p* = 0.023; ORc = 2.762; *p* = 0.036). Furthermore, we found a negative correlation between HAD-A scores and patient’s age.

**Conclusions:**

We pointed out through the current study the associated factors with anxiety and depression in patients under fertility management to precociously identify those who need psychological counseling and hence to better manage infertility issues.

## Introduction

Infertility is considered as a real public health problem because of its medical, socio-cultural and financial impact [1]. Its prevalence is estimated between 8 and 12% [2], which means that approximately one out of six couples consults for difficulties in conceiving during their reproductive life. In three quarters of cases, infertility is of male or female origin, or it associates both sexes [2]. Apart from its significant cost on society, infertility could have heavy psychological consequences on both partners, especially in a society where parenthood is one of the main objectives of life [3]. Indeed, the impossibility to conceive constitutes for those patients one of the most painful experiences of the couple life [4]. Starting from the investigation step (semen analysis, hormonal assessment…) until the announcement of infertility diagnosis and then infertility management, patients could have feelings of devaluation and decreased self-esteem [5, 6].

In some cultures, even undergoing semen analysis could be an emotionally hard experience as it could be perceived as a socially stigmatizing circumstance. In Tunisia, literature data on the topic are scarce. Indeed, only two studies describing the psychological impact of infertility on women [7] and on both partners [8] are available. None of these two studies has identified predictive factors of anxiety and/or depression in Tunisian population during fertility investigations.

For single patients, semen analysis could be prescribed to evaluate the potential impact of some urogenital pathologies (varicocele, hernia…) on semen quality and hence on future fertility potential. Masturbation in the laboratory could be perceived as a worrying situation for these young unmarried men. They always fear sperm collection failure which is considered as a shameful situation. Furthermore, they could have increasing concerns about their future sexual health. Examining mental health aspects in these teenagers is not an available topic among literature data until now.

Pointing out predictive factors of mental health alteration in these two categories of patients (married and unmarried) could be of great interest in precociously identifying patients who need psychological support during andrological investigation. This could help patients in better dealing with infertility issues as well as Assisted Reproductive Technologies (ART) partitioners in improving ART outcomes.

In this context of lack of literature data, the current study aimed to: (i) determine the level of anxiety and depression among Tunisian men during fertility related investigations and evaluate their effects on semen parameters. (ii) identify the predictive factors of anxiety and depression among those patients.

## Patients and methods

### Study design

This was a cross-sectional study performed in the Laboratory of Cytogenetics and Reproductive Biology of Fattouma Bourguiba University Teaching Hospital (Monastir, Tunisia) from August 2020 to March 2021.

### Study population

#### Inclusion criteria

Were included men addressed for semen quality assessment either in case of infertility (for those in couple) or to explore the impact of varicocele on semen parameters (for single patients). We also included male partners of couples addressed for Intra-Uterine Insemination (IUI).

#### Non-inclusion criteria

All men who had a previously diagnosed psychological disorder, those who presented severe depression and/or anxiety symptoms or had a stressful life event were not included in the current study.

### Data collection and operational definition of variables

Sociodemographic and medical data and information’s on lifestyle factors and infertility history were collected by a medical doctor at the Laboratory of Cytogenetics and Reproductive Biology of Fattouma Bourguiba University Teaching Hospital (Monastir Tunisia) after sperm collection, using a data collection sheet.

Among lifestyle factors, occupations that were considered to compromise fertility in the current study were those exposing to X-rays, high temperature, pesticides (farmers and gardeners), metals (metal workers and welders), chemical products (painters, varnishers, ceramics and paper industry workers) [9].

Patients reported their weight and height, and the body mass index (BMI) was calculated according to the standard formula of kg/m2. The patient was considered obese if the BMI was beyond 30.

### Psychological assessment

The subjects were evaluated for anxiety and depression symptoms using the valid Arab version of the HAD (Hospital Anxiety and Depression) scale [10]. It contains two 7-item sub-scales, one for anxiety and the other for depression. Each item in both scales has a range from 0 to 3. The used questionnaire was self-administrated for men on the day of semen collection and there was no one else present when the patient was answering the questionnaire in a quiet room.

HAD-D (Hospital Anxiety and Depression-Depression) and HAD-A Hospital Anxiety and Depression-Anxiety) scores were interpreted according to the following range: 0–7: no depression or anxiety disorder; 8–10: mild depression or anxiety disorder; 11–14: moderate disorder; and 15 and more: severe disorder.

When performing comparisons between patients with and without anxious or depressive symptoms, we considered in the current study that patients with a HAD-A or HAD-D score ranging between 0 and 10 as having no disorders and those having scores upper than 10 as having disorders in order to have comparable groups with regards to the number of included patients.

### Semen quality assessment

Semen samples were obtained by masturbation in the laboratory after 3 to 5 days of sexual abstinence. Sperm analysis was performed by a trained technician. Results interpretation weas performed according to 2021 WHO guidelines (World Health Organization) [11].

### Statistical analysis

Data analyses were performed using Statistical Package for Social Sciences for Windows version 21.0, (SPSS 21.0). The presentation of analyzed data was made through tabular methods and figures.

Baseline clinical characteristics and semen parameters were expressed as mean ± SD or median [interquartile range (IQR)] as appropriate for continuous numerical variables and as frequency (percentage) for categorical variables. Between-groups comparisons were performed with Student's t or Anova tests for numerical variables and a chi-squared test or Fisher's exact test for categorical variables.

Predictive factors of anxiety and depression were initially investigated via univariate analysis. Variables were compared with Chi^2^ Test. Odds Ratios (OR) were also determined; p values of 0.05 or less were considered statistically significant. Then, statistically significant factors as well as factors with a *p*-value between 0.05 and 0.20 were included in the binary logistic regression model to determine the independent predictive factors of anxiety and depression. The adjusted Odds Ratios (ORa) were also determined, and p values of 0.05 or less were considered statistically significant. Correlation analysis was done using Sperman’s correlation test.

### Ethical considerations

Informed consent was obtained from all the participants. They were given detailed information about the main purpose of the study and informed that the participation is voluntary, and the results are confidential. This study was approved by the ethics committee of the Faculty of Medicine of Monastir under the number IORG 0009738 N°115/ OMB 0990–0279.

## Results

### Study population

#### Baseline characteristics of the study population

All the solicited patients accepted to participate to the current study. There was no refusal to respond to the questionnaire.

The study population included a total of 282 patients whose mean age was 37 ± 6 years (IQR [33–41]) with extremes varying from 17 to 63 years old. Baseline characteristics are detailed in Table 1.

**Table 1:** Baseline characteristics of men undergoing infertility investigation or treatment

**Variable**	**Effective** (n)	**Percentage (%)**
**Age class**		
< 20	3	**1.06**
20 – 34	92	**32.62**
35 – 44	162	**57.44**
> = 45	25	**8.86**
**Tobacco**		
No	112	**39.70**
Yes	170	**60.30**
**Alcohol**		
No	225	**79.80**
Yes	57	**20.20**
**Obesity**		
No	34	**12.05**
Yes	137	**48.58**
NA^a^	111	**39.36**
**Compromising fertility occupation**		
No	210	**74.50**
Yes	72	**25.50**
**Marital Status**		
Single	10	**3.50**
Married	272	**96.50**
**Pathological History**		
**Medical**		
No	256	**95.20**
Yes	26	**4.80**
Diabetes	3	**1.10**
Kidney disease	6	**2.10**
Dysthyroid	1	**0.40**
Inflammatory Bowel Disease	2	**0.70**
Others	14	**0.50**
**Surgical**		
No	270	**4.30**
Yes	12	**95.70**
**Urogenital**		
No	219	**77.60**
Yes	63	**22.40**
Varicocele	39	**13.80**
Inguinal Hernia	5	**1.80**
Testicular torsion/trauma	6	**2.10**
Urogenital Infection	4	**1.40**
Mumps	1	**0.40**
Testicular Ectopia	5	**1.80**
Erectile Dysfunction	3	**1.10**

^a^*NA* Not Assessed

### Infertility characteristics

A total of 10 single patients and 272 married ones were addressed for semen quality assessment.

Infertility characteristics are detailed in Table 2.

**Table 2:** Infertility characteristics in men undergoing infertility investigation or treatment

Features	Number of Patients (n)	Percentage (%)
**Infertility Origin**		
Male	181	65.54
Female	12	4.41
Both	89	31.50
**Marital Status/Infertility character**		
Single	10	3.50
Primary Infertility	189	67.10
Secondary Infertility	83	29.40
**Infertility duration in months (Median, IQR**^**a**^** [25%—75%])**	24 [12—48]	

^a^Inter-Quartile Rang

### Semen parameters

Semen parameters are detailed in Table 3.

**Table 3:** Semen analysis results in men undergoing infertility investigation or treatment

Parameters	Number of samples (n)	Percentage (%)
**Semen volume**		
Normal	236	**83.70**
Abnormal	46	**16.30**
**Sperm count**		
Normal	224	**79.40**
Abnormal	58	**20.60**
**Total sperm motility**		
Normal	61	**21.60**
Abnormal	221	**78.40**
**Progressive sperm motility**		
Normal	49	**17.40**
Abnormal	231	**81.90**
NA^a^	2	**0.70**
**Sperm vitality**		
Normal	173	**61.30**
Abnormal	25	**8.90**
NA^a^	84	**29.80**
**Sperm morphology**		
Normal	25	**8.90**
Abnormal	174	**61.70**
NA^a^	83	**29.40**
**MAI**^b^		
Normal	2	**0.70**
Increased	197	**69.90**
NA^a^	83	**29.40**
**Sperm viscosity**		
Normal	267	**94.70**
Abnormal	15	**5.30**
**Leucocytospermia**		
No	249	**88.30**
Yes	33	**11.70**

^a^*NA* Not Assessed

^b^*MAI* Multiple Abnormalities Index

### Anxiety and depression evaluation

**Fig. 1:**
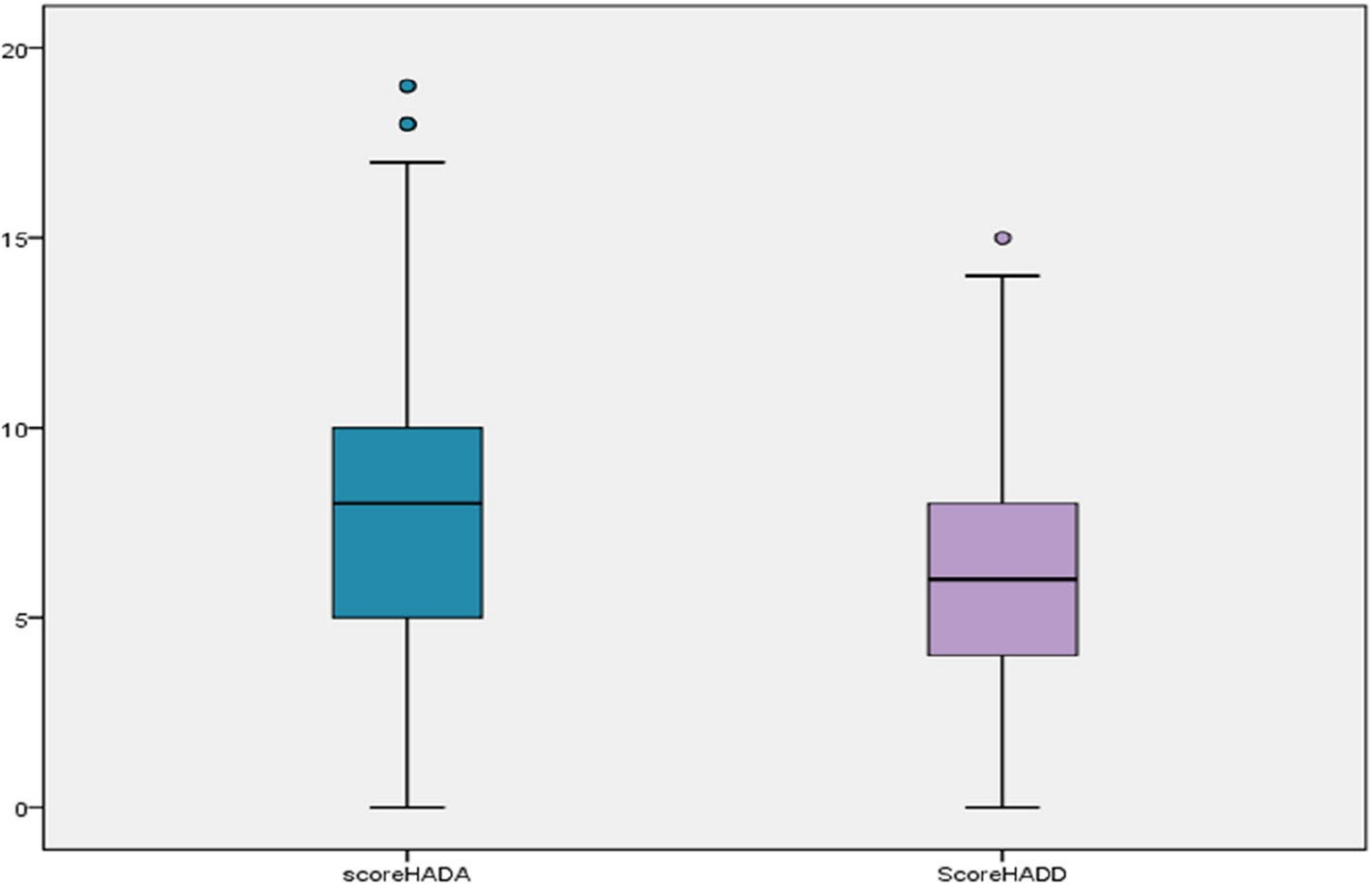
Box plot of anxiety and depression scores (HAD-A, HAD-D)

The mean HAD-D (depression) and HAD-A (anxiety) scores were of 6.56 ± 3.07 (IQR [4–8]) and 7.94 ± 3.73 (IQR [5–10]) respectively. The box plot below (Fig. 1) shows the distribution of the two assessed scores among the studied population.

**Fig. 2:**
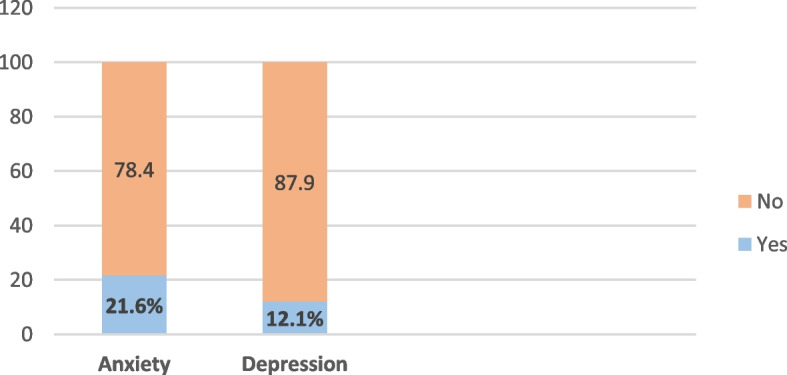
Anxiety and depression among the studied population

As shown in Fig. 2, anxious symptoms were recorded in 21.60% of the population whereas 12.10% of patients exhibited depressive symptoms.

HAD-A and HAD-D scores according to demographic data and infertility characteristics of the included patients are shown in Table 4.

**Table 4:** Anxiety and depression scores among men undergoing infertility investigation or treatment according to demographic data and infertility characteristics

Factors	Anxiety		Depression	
	**HAD-A score (mean ± SD)**	***p*-value**	**HAD-D score (mean ± SD)**	***p*-value**
**Age class(years)**				
< 20	13.67 ± 4.51	**0.020**	6 ± 3.60	0.759
20 – 34	8.38 ± 3.61		6.34 ± 3.00	
35 – 44	7.69 ± 3.66		6.63 ± 3.06	
> = 45	7.32 ± 4.05		7 ± 3.40	
**Tobacco**				
No	7.52 ± 3.95	0.128	6.31 ± 3.11	0.274
Yes	8.22 ± 3.56		6.72 ± 3.04	
**Alcohol**				
No	7.73 ± 3.66	0.176	6.44 ± 3.11	0.159
Yes	8.77 ± 3.89		7.05 ± 2.88	
**Obesity**				
No	7.74 ± 3.70	0.465	6.49 ± 2.94	0.289
Yes	7.26 ± 3.32		5.97 ± 2.42	
**Infertility duration (months)**				
< 6	7.29 ± 3.50	0.670	5.00 ± 2.24	**0.011**
6 – 12	7.74 ± 3.49		5.91 ± 2.72	
> = 12	7.98 ± 3.75		6.79 ± 3.13	
**Infertility origin**				
Male	7.99 ± 3.78	0.920	6.52 ± 2.89	0.925
Female	8.17 ± 3.12		6.42 ± 3.23	
Both	7.82 ± 3.72		6.66 ± 3.41	
**Marital Status/Infertility character**				
Single	9.50 ± 4.55	0.344	7.50 ± 3.24	0.250
Primary infertility	7.80 ± 3.65		6.36 ± 2.88	
Secondary infertility	8.08 ± 3.80		6.90 ± 3.43	

Are shown in bold *p*-values <  = 0.05

*SD* Standard Deviations

### Predictive factors of anxiety and depression

#### Predictive factors of anxiety

Results of univariate analysis investigating the possible anxiety determinant factors among baseline characteristics are detailed in Table 5.

**Table 5:** Predictive factors of anxiety among men undergoing infertility investigation or treatment, univariate analysis. Univariate analysis of demographic and lifestyle factors associated with anxiety

Variable	Anxiety (*n* = 61)	Total (*N* = 282)	Univariate analysis		
	**n (%)**	**N**	**ORc**^**a**^	**CI**^**b**^** 95%**	***p*-value**
**Age (years)**					
< 45	55 (**21.40**)	257	1.160	[0.442- 3.044]	0.763
> = 45	6 (**24**)	25			
**Single**					
No	56 (**20.60**)	272	3.857	[1.079–13.789]	0.068
Yes	5 (**50**)	10			
**Smoking**					
No	22 (19.60)	112	1.218	[0.677- 2.192]	0.510
Yes	39 (22.90)	170			
**Alcohol**					
No	44 (**19.60**)	225	1.748	[0.907- 3.370]	0.093
Yes	17 (**29.80**)	57			
**Compromising fertilityoccupation**					
No	42 (**20**)	210	1.434	[0.769- 2.657]	0.256
Yes	19 (**26.40**)	72			
**Number of children**					
0	51 (**20.90**)	244	1.352	[0.616- 2.964]	0.451
> = 1	10 (**26.30**)	38			
**Obesity**					
No	29 (**21.20**)	137	0.642	[0.228- 1.805]	0.398
Yes	5 (**14.70**)	34			
**Urogenital history**					
No	44 (**20.10**)	219	1.470	[0.770- 2.807]	0.242
Yes	17 (**27**)	63			
**Medical history**					
No	53 (**20.70**)	256	2.730	[0.835- 8.925]	0.235
Yes	8 (**30.80**)	26			
**Surgical history**					
No	56 (**20.70**)	270	1.470	[0.770- 2.807]	0.172
Yes	5 (**41.70**)	12			
**Number of comorbidities**					
0 -1	55 (**20.30**)	271	4.713	[1.387–16.014]	**0.007**
> = 2	6 (**54.50**)	11			
**Spermogram rank**					
1	30 (**23.40**)	128	0.823	[0.467- 1.453]	0.502
> = 2	31 (**20.10**)	154			
**Recurrent miscarriage history**					
< 3	58 (**21.20**)	274	2.234	[0.519- 9.626]	0.269
> = 3	3 (**37.50**)	8			
**ART attempts failure**					
No	52 (**20.80**)	250	1.490	[0.650- 3.413]	0.343
Yes	9 (**28.10**)	32			
**Infertility duration**					
< 12 months	12 (**20.70**)	58	1.073	[0.528- 2.183]	0.845
> = 12 months	49 (**21.90**)	224			
**Infertility origin**					
Female	4 (**33.30**)	12	0.535	[0.156- 1.841]	0.517
Male or both	57 (**21.10**)	270			
**Marital status/Infertility character**					
Primary or secondary	56 (**20.60**)	272	3.857	[1.079–13.789]	**0.027**
Single	5 (**50**)	10			

Are shown in bold *p*-values <  = 0.05

^a^Crude Odds Ratio

^b^Confidence Interval

Univariate analysis results focusing on the possible links between standard semen parameters and anxiety symptoms are shown in Table 6.

**Table 6:** Predictive factors of anxiety among men undergoing infertility investigation or treatment, univariate analysis. Univariate analysis of semen parameters associated with anxiety

Parameters	Anxiety (*n* = 61)	Tota (*N* = 282)	Univariate analysis		
	**n (%)**	**N**	**ORc**^**a**^	**CI**^**b**^ **95%**	**p**
**Semen abnormalities**					
No	1 (9.10)	11	2.844	[0.357- 22.660]	0.511
Yes	60 (22.10)	271			
**Number of semen abnormalities**					
0—1	21 (20.20)	104	1.146	[0.632- 2.075]	0.654
> = 2	40 (22.50)	178			
**Total sperm motility**					
Normal	11 (18)	61	1.329	[0.644- 2.744]	0.441
Abnormal	50 (22.60)	221			
**Progressivesperm motility**					
Normal	10 (19.60)	51	1.162	[0.544- 2.479]	0.698
Abnormal	51 (22.10)	231			
**Sperm Morphology**					
Normal	26 (22.20)	117	0.942	[0.531- 1.673]	0.839
Abnormal	35 (21.20)	165			
**Necrozoospermia**					
No	55 (21.40)	257	1.160	[0.442- 3.044]	0.763
yes	6 (24)	25			
**Leucospermia**					
No	52 (20.90)	249	1.421	[0.623- 3.241]	0.402
Yes	9 (27.30)	33			
**Increased MAI**					
No	19 (22.40)	85	0.941	[0.510- 1.739]	0.874
Yes	42 (21.30)	197			
**Sperm count**					
Normal	48 (21.40)	224	1.059	[0.529- 2.122]	0.871
Abnormal	13 (22.40)	58			
**Azoospermia**					
No	58 (21.20)	274	2.234	[0.519- 9.626]	0.503
Yes	3 (37.50)	8			
**Semen volume**					
Normal	50 (19.80)	252	2.339	[1.046- 5.229]	**0.034**
Abnormal	11 (36.70)	30			

Are shown in bold *p*-values <  = 0.05

^a^ Crude Odds Ratio

^b^Confidence Interval

Multivariate analysis showed that only cumulative comorbidities was an independent predictive factor of anxiety in the studied population (Table 7).

**Table 7:** Independent factors of anxiety among men undergoing infertility investigation or treatment, multivariate analysis

Variable	OR_a_^a^	CI^b^ 95%	*p*-value
**Cumulative comorbidities**			
0 or 1	Ref		
> = 2	3.74	[1.02–13.63]	**0.046**

Are shown in bold *p*-values <  = 0.05

^a^Adjusted Odds Ratio

^b^Confidence Interval

### Predictive factors of depression

Details of univariate analysis are shown in Table 8 for baseline characteristics and in Table 9 for semen parameters.

**Table 8:** Predictive factors of depression among men undergoing infertility investigation or treatment, univariate analysis. Univariate analysis of demographic and lifestyle factors associated with depression

Features	Depression (*N* = 97)	Total (*N* = 282)	Univariate analysis		
	**n (%)**	**N**	**ORc**^**a**^	**CI**^**b**^** 95%**	***p*-value**
**Age (years)**					
< 45	29 (11.30)	257	1.966	[0.685–5.636]	0.339
> = 45	5 (20)	25			
**Single**					
No	31 (11.40)	272	3.332	[0.819–13.555]	0.201
Yes	3 (30)	10			
**Smoking**					
No	11 (9.80)	112	1.437	[0.671- 3.078]	0.349
Yes	23 (13.50)	170			
**Alcohol**					
No	25 (11.10)	225	1.500	[0.658- 3.421]	0.333
Yes	9 (15.80)	57			
**Compromising fertility occupation**					
No	21 (10)	210	1.983	[0.936- 4.202]	0.070
Yes	13 (18.10)	72			
**Number of children**					
0	27 (11.10)	244	1.815	[0.729- 4.520]	0.304
> = 1	7 (18.40)	38			
**Obesity**					
No	16 (11.70)	137	0.473	[0.103- 2.163]	0.501
Yes	2 (5.90)	34			
**Urogenital history**					
No	30 (13.70)	219	0.427	[0.145- 1.262]	0.114
Yes	4 (6.30)	63			
**Medical history**					
No	28 (10.90)	256	2.443	[0.905- 6.595]	0.135
Yes	6 (23.10)	26			
**Surgical history**					
No	31 (11.50)	270	2.570	[0.660- 10.004]	0.340
Yes	3 (25)	12			
**Number of comorbidities**					
0 -1	31 (11.40)	271	2.903	[0.731- 11.524]	0.268
> = 2	3 (27.30)	11			
**Spermogram rank**					
0- 1	12 (9.40)	128	1.611	[0.764- 3.398]	0.207
> = 2	22 (14.30)	154			
**Recurrent miscarriage history**					
< 3	29 (13.60)	213	0.496	[0.184- 1.335]	0.158
> = 3	5 (7.20)	69			
**ART attempts failure**					
No	31 (12.40)	250	0.731	[0.210- 2.543]	0.836
Yes	3 (9.40)	32			
**Infertility duration**					
< 12 months	1 (1.70)	58	9.848	[1.318 – 73.596]	**0.007**
> = 12 months	33 (14.70)	224			
**Infertility origin**					
Female	2 (16.70)	12	1.488	[0.312 – 7.096]	0.962
Male or both	32 (11.90)	270			
**Marital status/Infertility character**					
Primary or secondary	31 (11.40)	272	3.332	[0.819- 13.555]	0.201
Single	3 (30)	10			

Are shown in bold *p*-values <  = 0.05

^a^ Crude Odds Ratio

^b^Confidence Interval

**Table 9:** Predictive factors of depression among men undergoing infertility investigation or treatment, univariate analysis. Univariate analysis of semen parameters associated with depression

	**Depression (*N* = 97)**	**Total (*N* = 282)**	**Univariate analysis**		
	**n (%)**	**N**	**ORc**^**a**^	**CI**^**b**^** 95%**	***p*-value**
**Semen abnormalities**					
No	2 (18.20)	11	0.603	[0.125- 2.913]	0.870
Yes	32 (11.80)	271			
**Number of semen abnormalities**					
0—1	7 (6.70)	104	2.478	[1.039 – 5.911]	**0.036**
> = 2	27 (15.20)	178			
**Total spermmotility**					
Normal	5 (10.0)	50	1.074	[0.443 – 2.600]	0.875
Abnormal	29 (12.50)	232			
**Progressive sperm motility**					
Normal	4 (7.80)	52	1.754	[0.589- 5.219]	0.307
Abnormal	30 (13)	230			
**Sperm morphology**					
Normal	8 (6.80)	117	2.549	[1.110- 5.851]	**0.023**
Abnormal	26 (15.80)	165			
**Necrozoospermia**					
No	31 (12.10)	254	0.994	[0.281 – 3.516]	0.993
yes	3 (12)	28			
**Leucospermia**					
No	29(11.60)	249	1.355	[0.485- 3.784]	0.767
Yes	5(15.20)	33			
**Increased MAI**					
No or NA^c^	5 (5.90)	65	2.762	[1.031- 7.401]	**0.036**
Yes	29 (14.70)	217			
**Sperm count**					
Normal	76 (33.50)	227	0.808	[0.318- 2.054]	0.653
Abnormal	21 (38.20)	55			
**Azoospermia**					
No	34 (12.40)	274	0.876	[0.838- 0.916]	0.609
Yes	0 (0)	8			
**Semen volume**					
Normal	28 (11.90)	252	1.114	[0.433- 2.865]	0.822
Abnormal	6 (13)	30			

Are shown in bold *p*-values <  = 0.05

^a^Crude Odds Ratio

^b^Confidence Interval

^c^Not Assessed

Multivariate analysis showed that being single (OR_a_ = 7.20; *p* = 0.035), having a compromising fertility occupation (OR_a_ = 2.35; *p* = 0.044), and having urogenital pathological history (OR_a_ = 4.90; 0.023) were independents risk factors of depression (Table 10).

**Table 10:** Independents factors of depression among men undergoing infertility investigation or treatment, multivariate analysis

Variable	OR_a_^*^	CI^**^ 95%	*p*-value
**Single**			
No	Ref		
Yes	7.20	[1.14–45.63]	**0.035**
**Compromising fertility occupation**			
No	Ref		
Yes	2.35	[1.02- 05.41]	**0.044**
**Urogenital history**			
No	Ref		
Yes	4.90	[1.24- 19.33]	**0.023**

Are shown in bold *p*-values <  = 0.05

^*^Adjusted Odds Ratio

^**^Confidence Interval

### Correlation study

**Fig. 3:**
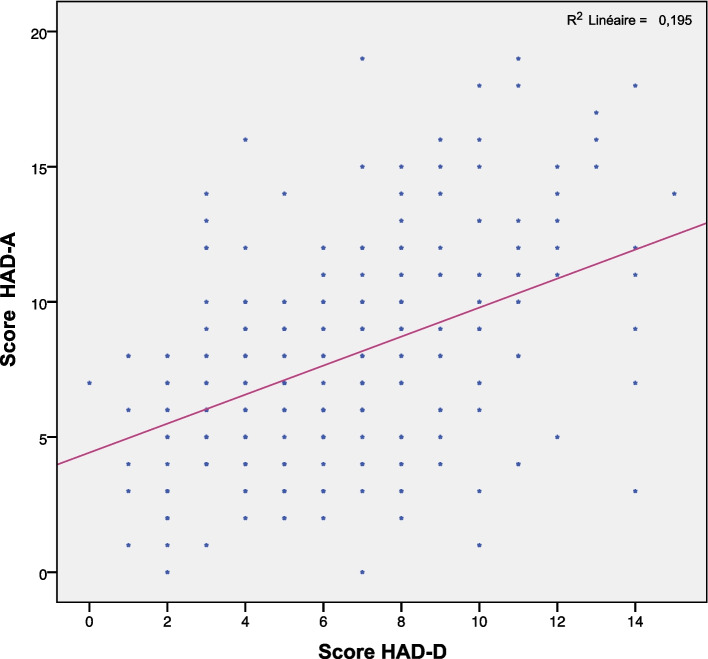
Scatter plot between HAD-D score and HAD-A score

We have noticed a positive correlation between anxiety and depression scores (R = 0.44; *p* < 0.001). The link between these parameters is shown in Fig. 3.

**Fig. 4:**
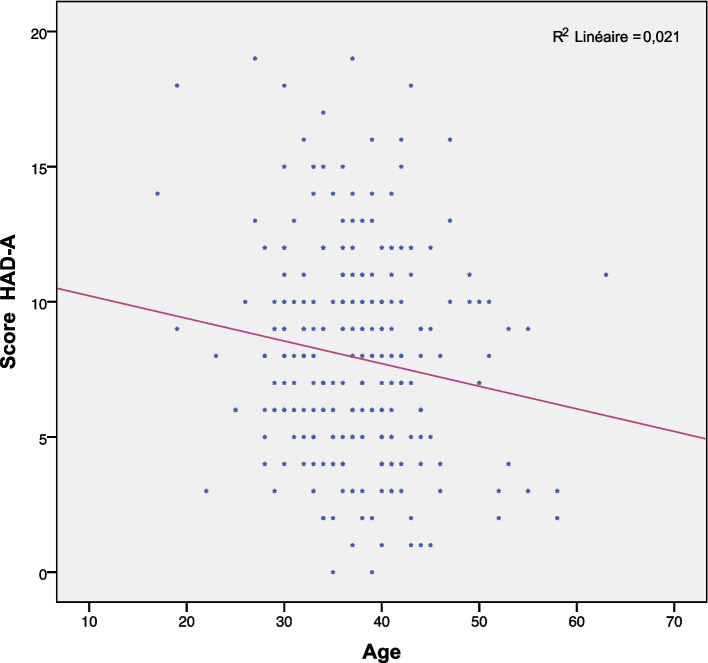
Scatter plot between age and HAD-A score

Besides, HAD-A scores were negatively correlated with age (R = -0.14; *p* = 0.015) as shown in Fig. 4.

No further correlations were found between depression as assessed by HAD-D score and the remaining studied parameters.

## Discussion

This study showed that single patients presented higher levels of anxiety when compared to married ones. Furthermore, patients with two or more comorbidities were more anxious than others. Having at least two comorbidities was also pointed out as an independent factor of anxiety in the studied population.

We have shown in the other hand, that, patients were more vulnerable to develop depressive symptoms as the duration of infertility increases. Being single, having urogenital pathologies as well as having an occupation known to compromise male fertility were highlighted as independent factors of depression in the current study.

In many countries such as Tunisia, little attention is paid to the psychological well-being of patients when dealing with infertility issues. The medical staff is often focusing on the investigations to do, the ovarian stimulation protocol to indicate and the appropriate ART technique to perform. The literature on the mental health of Tunisian men with infertility is scarce. The only published study is that of El Kissi and collaborators who, aimed to compare the psychological profiles of the two partners in a total of 100 infertile couples in 2013 [8].

Studying the link between psychological stress and fertility issues is attracting increasing interest due to the increasing prevalence of stressful lifestyle worldwide. Literature data are mainly focusing on the psychological profile of the female partner who undergoes the majority of treatments during infertility management course [7, 12–14].

In some countries, dealing with infertility could be psychologically harder for the male partner due to cultural, social and religious reasons.

We only enrolled patients without diagnosed psychological diseases and acute stressful life event to avoid introducing any bias to the obtained results.

When focusing on the subgroup of single patients, they were all of them addressed to investigate the potential impact of varicocele on semen parameters. According to the literature data, the incidence of varicocele in adolescent aged 15–19 years is about 15%. It increases to reach 35–44% in adults with primary infertility and is about 45–81% in case of secondary infertility [15].

With regards to semen quality, despite the respect of the recommended sexual abstinence delay, normal semen parameters were observed in only 8 patients out of 282 (2.83%). Except from some parameters which were normal in more than half of the cases (semen volume, viscosity, sperm count and vitality, leukocytospermia), we have shown impairment in sperm motility (both progressive and total) and morphology. The MAI was also beyond the WHO thresholds in roughly 70% of cases. It’s of great importance to mention that the study population included either child-seeking patients or those addressed to evaluate the impact of urogenital pathologies (mainly varicocele) on semen quality of single adolescents. This could explain the high proportion of abnormal semen quality among the assessed samples. Moreover, the above-mentioned lifestyle factors could once again play a key role in the observed alterations.

When focusing on the mean values we can conclude that levels of anxiety and especially those of depression were not really elevated in our population. These moderate levels of anxiety and depression could be explained by mainly two reasons: when patient is addressed to our lab or have an appointment for semen analysis, he is provided with all necessary explanations about the required conditions for semen collection and the different parameters to be assessed. By providing these explanations, we aim to involve the patient in the therapeutic care process. Hence, he doesn’t feel only to undergo infertility investigations and treatments, but he is one of the main actors of that process. Besides, as the questionnaire is answered on the day of semen collection (before confronting patients with results), the men presenting for semen analysis are usually healthy men, who might also believe that they have no fertility problems.

Even if they were moderate, levels of anxiety and depression were shown to be positively correlated. Such a correlation confirms the interest of assessing these two parameters in investigating the mental health of the included patients.

Anxiety levels were shown to be the highest in patients under 20 years old and the lowest in those aged over 54 years old. Younger patients are more anxious about their future fertility especially if they are aware of the risk of a further possible increase in semen quality impairment with age. Indeed, it was more common to consider advanced maternal age as one of the main factors of couple infertility because of an altered oocyte quality [16, 17]. However, several studies have recently shown that male age could also be a real threat to semen quality and sperm DNA integrity [18, 19]. This is particularly to consider in societies where parenthood at an older age is becoming a trend among men [20]. Taking into account the above considerations, the observed high level of anxiety in patients under the age of 20 years old, is likely to increase with age and especially when having the parenthood project. The results of correlation study have shown a negative correlation between HAD score and male age. Older men are less anxious about their fertility potential.

With regards to depression related symptoms, we observed a significant increase in patients being infertile for 12 months and more. This delay corresponds to that fixed by the WHO to define infertility. These results underlie the evolutionary character of depressive symptoms in infertile patients. In a recent study [21], the increase in infertility duration was also identified as a risk factor for the occurrence of psychological distress and sexual dysfunction in infertile women.

Having comorbidities could be a source of feelings of worthlessness and guilt since this category of patient would attribute their difficulties in conceiving to the chronic pathology they present. A cross sectional study including 1215 Danish men has concluded that patients with comorbidities in the reproductive sphere are significantly more stressed than others [22].

Single patients presented higher levels of anxiety when compared to married ones. Similar results were reported in a recent study conducted during the COVID-19 pandemic [23]. Single patients are more worried about their ability of conceiving especially that they are not supported by the partner. Companionship and attention from the partner are known to play a key role in coping with psychological distress and infertility adjustment [23–25].

The effect of distress in worsening male fertility has been investigated in some previous reports [22, 26]. Vellani and collaborators [26] have shown for the first time in 2013 an association between both state and trait anxiety with semen quality impairment (semen volume, sperm count and total motility). A recent study including 378 infertile men who were evaluated for anxiety levels have demonstrated [27]. These findings underline a possible role of neuroendocrine factors in altering the spermatogenesis process. Exposure to stress could inhibit the hypothalamic-pituitary–gonadal axis and induce a decrease in testosterone level which directly impacts semen quality through a reduction in semen volume, sperm count and motility [28, 29]. In the same trend, Bhongade and colleagues have noticed that patients with increased HAD scores have reduced sperm count, motility and morphologically normal spermatozoa [30].

In a recent study including 391 men, Ye and al found that men with depression had worse semen quality parameters, including semen volume, sperm concentration, total sperm count, total motility, and progressive motility [31].

These findings were documented at the hormonal level by a decrease in testosterone level and an increase in LH and FSH levels in the studied population. Besides, psychological stress was shown to be associated with a decrease in seminal antioxidants and hence with higher levels of seminal oxidative stress [32]. The imbalance between seminal plasma antioxidant properties and reactive oxygen species (ROS) is harmful for spermatogenesis and could explain the observed alteration in sperm morphology as seen in the current study.

Being single was identified as risk factor of both anxiety and depression in our population. Single patients are faced to greater difficulties in coping with depression and anxiety.

Pointing out a detrimental effect of occupation on the psychological profile of hypofertile patients draws attention to the negative impact of professional exposure on the mental health of our patients. This supposes that patients are aware of the harmful impact of their profession on the fertility potential. It is well established that exposure to chemical compounds termed endocrine disrupting chemicals (EDCs), could act as anti-androgen and deregulate spermatogenesis [33]. A large Tunisian study including 2122 men has established the link between EDCs and semen quality impairment [9].

Interestingly, embarking on IUI (Intra Uterine Insemination) treatment wasn’t an associated factor neither with anxiety nor with depression symptoms in our population. This could be mainly explained by two reasons: (i) these patients might see the treatment as hope for solving their difficulty to conceive, (ii) they have already performed several infertility investigations and they are informed about the details of their treatment course.

To the best of our knowledge, this was the first Tunisian and Nord African study to investigate anxiety and depression levels of patients under andrological investigation. The current study advanced current knowledge about anxiety and depression profiles of a large population of hypofertile Tunisian patients. Our results have pointed out categories of patients who were more vulnerable to anxiety and/or depression. Screening these patients could be of great interest because it allows referring them precociously to psychologist and so helping them to manage anxiety and/or depression.

Among multiples available questionnaires measuring anxiety and depression levels, we used the Arab version of the HAD scale. As it is used as a self-administered questionnaire, potential interviewer-biases were avoided. However, for ethical reasons mainly related to a possible greater risk of semen collection failure when administering the questionnaire before semen collection, this latter was given to the patient just after semen collection.

Semen analysis was performed by the same experimented lab technician for all the samples. Data interpretation was initially performed according to 2010 WHO guidelines [34] and has been redone after the publication of the latest version of the WHO guidelines in July 2021 [11] in order to be updated.

It bears noting that our study has some limitations. The current study was conducted between August 2020 and March 2021. As the severe acute respiratory syndrome coronavirus 2 (SARS-COV-2) emerged on March 2020 in Tunisia, we cannot exclude a possible impact of COVID-19 pandemic context on the psychological profile of patients.

Furthermore, despite of the large number of included patients, the size of the subgroup of single patients remains restricted. A larger sample of single patients having urogenital pathologies indicating spermogram analysis would better help understanding the psychological profile of this category of patients.

Furthermore, investigating the possible explanation of impaired semen quality by assessing hormone’s levels (LH, FSH and testosterone) and evaluating the level of oxidative stress in the seminal plasma of anxious and depressive patients would be of great interest in elucidating the implicated pathways leading to semen quality impairment. Seminal oxidative stress assessment in the studied population is currently in process and would provide more data on the topic to answer the question whether anxiety and/or depression related symptoms are the mirror of a seminal oxidative stress in these patients.

## Conclusion

This is, to the best of our knowledge, the first study to evaluate the psychological profile of a large population of patients on the day of semen analysis in Tunisia and North Africa.

Our study has concluded that levels of anxiety and especially those of depression were moderate in our population.

Taken together, our results mainly demonstrated that patients with two or more comorbidities are more prone to develop anxiety and that single patients, those having urogenital pathologies as well as those working in a reprotoxic environment are more likely to be depressed. Hence, these categories of patients could be identified at the beginning of infertility course (or fertility investigation in case of single patients) to be provided with the appropriate psychological care.

With regards to semen parameters, patients with hypospermia were more anxious compared with those with normal semen volume. As for depression, we observed that patients exhibiting two or more semen abnormalities, teratozoospermia and increased MAI were more likely to be depressed.

Awareness and recognition of predictive factors of anxiety and depression and their impact on semen parameters of men under hypo fertility investigation is a crucial step before providing these patients with the appropriate support.

Based on the results of the current study, implementing a psychiatry or psychology consultation in Tunisian andrology as well as ART laboratories seems to be mandatory. This is already the case in many laboratories in the developed countries but not yet in Tunisia. Patients under andrological evaluation or ART treatment express distinct need for emotional support and so require individualized psychological care.

## Data Availability

The datasets generated during the current study are available from the corresponding author on reasonable request.

## Abbreviations

ART: Assisted Reproductive Technology
BMI: Body Mass Index
DNA: Deoxyribonucleic acid
EDC: Endocrine Disturbing Chemicals
HAD: Hospital Anxiety and Depression
IUI: Intra-uterin Insemination
MAI: Multiple Abnormalities Index
ROS: Reactive Oxygen Species
WHO: World Health Organization
FSH: Follicle Stimulating Hormone
LH: Luteizing Hormone
HAD-D: Hospital Anxiety and Depression-Depression
HAD-A: Hospital Anxiety and Depression-Anxiety
